# A Quantile Mapping Bias Correction Method Based on Hydroclimatic Classification of the Guiana Shield

**DOI:** 10.3390/s17061413

**Published:** 2017-06-16

**Authors:** Justine Ringard, Frederique Seyler, Laurent Linguet

**Affiliations:** 1 Université de Guyane, UMR ESPACE-DEV, IRD, Université de La Réunion, Université de Montpellier, F-97300 Cayenne, French Guiana; linguetlaur@gmail.com; 2 IRD, UMR ESPACE-DEV, Université de Guyane, Université de La Réunion, Université de Montpellier, Maison de la Télédétection, 500 rue Jean-François Breton, F-34093 Montpellier CEDEX 5, France; frederique.seyler@ird.fr

**Keywords:** quantile mapping bias correction, hydroclimatic area, temporal distribution, TRMM-TMPA 3B42V7, Guiana Shield

## Abstract

Satellite precipitation products (SPPs) provide alternative precipitation data for regions with sparse rain gauge measurements. However, SPPs are subject to different types of error that need correction. Most SPP bias correction methods use the statistical properties of the rain gauge data to adjust the corresponding SPP data. The statistical adjustment does not make it possible to correct the pixels of SPP data for which there is no rain gauge data. The solution proposed in this article is to correct the daily SPP data for the Guiana Shield using a novel two set approach, without taking into account the daily gauge data of the pixel to be corrected, but the daily gauge data from surrounding pixels. In this case, a spatial analysis must be involved. The first step defines hydroclimatic areas using a spatial classification that considers precipitation data with the same temporal distributions. The second step uses the Quantile Mapping bias correction method to correct the daily SPP data contained within each hydroclimatic area. We validate the results by comparing the corrected SPP data and daily rain gauge measurements using relative RMSE and relative bias statistical errors. The results show that analysis scale variation reduces rBIAS and rRMSE significantly. The spatial classification avoids mixing rainfall data with different temporal characteristics in each hydroclimatic area, and the defined bias correction parameters are more realistic and appropriate. This study demonstrates that hydroclimatic classification is relevant for implementing bias correction methods at the local scale.

## 1. Introduction

Given the current context of climate change, it is essential to improve our understanding of the spatial and temporal dynamics of precipitation on the global and regional scales. Precipitation is an essential part of the global water cycle and its measurement is particularly crucial. Observation of precipitation at high spatial resolution is very important for monitoring and forecasting extreme weather events such as floods and droughts, but also for obtaining input data for hydrological applications and climate studies. However, obtaining accurate gauge-based precipitation measurements at high spatial resolution is difficult because of various technical and practical limitations [1]. Technical limitations can be exposed during heavy rainfall when water can accumulate at a rate faster than can be cleared by the calibration trough of the measurement device. Conversely, during light rainfall, water can evaporate from the collector. Practical limitations arise from the challenges associated with the installation and maintenance of a dense network of measurement devices in areas that are difficult to access, such as mountains, deserts, and primary forests [1,2].

Satellite precipitation products (SPPs) such as the Tropical Rainfall Measuring Mission Multisatellite Precipitation Analysis (TRMM-TMPA 3B42) [3,4,5], Climate Prediction Center MORPHing (CMORPH) product [6], and Precipitation Estimation from Remotely Sensed Information using Artificial Neural Networks (PERSIANN) [7] provide alternatives for obtaining precipitation data in regions with an insufficient distribution of rain gauge measurement stations. These SPPs with high spatial resolution (0.25° × 0.25°) and high temporal resolution (3 h) [8,9,10,11,12,13,14,15] are produced using data obtained by different instruments deployed on several satellites. The algorithms of these products link cloud brightness temperature, measured in the infrared band, with data relating to the size and characteristics of hydrometeors, as measured by microwaves [1,14,16].

All the above blended rainfall products are subject to different types of error depending on the quality of the measurements made by the sensor as well as the climate, topography, season, and local climatic regime. Several studies have addressed SPP failures in different geographic regions based on the intensity of precipitation and/or seasonal precipitation. The first evident defect of SPPs is that they overestimate low daily intensities (<2 mm/day) and underestimate high daily intensities (>20 mm/day) [8,11,12,13,14,17,18,19]. A second defect, observed by many authors, is that SPP performance depends on season, with greater errors detected in winter compared with summer or rainy seasons [9,10,12,18,20,21,22,23,24,25]. Ebert et al. [24] showed that as the precipitation regime tends toward deep convection, the accuracy of the satellite estimates improves. A third defect observed in SPPs is that their effectiveness depends on the climatic regime and that they show poorer performance in the driest areas [12,13,17,26]. A fourth defect relates to the difference in performance of SPPs according to topography, with low efficiency reported for SPPs in mountainous regions [9,17,27,28,29]. Finally, SPPs have a low ability to detect the daily precipitated volume [27,30,31]. Indeed, SPPs are less able to detect daily rainfall volume than rainfall occurrence. To correct all these defects, bias adjustment is essential prior to the use of SPPs in hydrological applications.

Different bias adjustment approaches are used to improve the quality of SPP data. The linear correction method corrects the average precipitation value based on the differences between the rain gauge data and satellite data. However, this method does not correct the variance and all events are adjusted with the same correction factor [15,32,33,34,35,36,37]. The Local Intensity Scaling method combines a precipitation threshold with linear scaling [32,37,38]. This method separately corrects wet-day frequency and wet-day intensity, applied pointwise and individually for each day of the year, and the estimated precipitation is corrected using a scaling factor. Nevertheless, the results obtained with this method are limited because, as with linear correction, the standard deviation is not corrected and all events are adjusted using the same correction factor. The Power Transformation method corrects the mean and variance of the temporal series of estimated precipitation [37,39,40,41]. This is a nonlinear correction in an exponential form that combines the correction of the coefficient of variation with linear scaling. The coefficient of variation of both daily and multiple-day precipitation amounts depends on the wet-day frequency, but this correction does not adjust the frequency of wet days [40]. The Quantile Mapping method (QM) [32,37,39,42,43,44,45,46,47,48,49,50,51,52], also named Distribution Mapping or the Quantile–Quantile method, adjusts the cumulative distribution of estimated data to the cumulative distribution of rain gauge data using a transfer function. This correction can capture the evolution of the mean and the variability of precipitation while matching all statistical moments. Most of these methods use rain gauge data to correct SPP data located in the same location (with respect to the satellite pixel). The aim of this article is to correct the SPP data for the Guiana Shield using a novel two set approach, pixel by pixel, without taking into account the daily rain gauge data of the pixel to be corrected, but instead using the daily rain gauge data of the surrounding pixels. We introduce the concept of spatial scale change analysis. The concept of scale must be involved in any spatial statistical analysis.

All the above bias correction methods are used to correct SPP estimates to provide results that are acceptable on the global scale; however, these methods are limited when correcting SPPs on the local scale. One limitation of applying these methods on the global scale is that precipitation estimates are corrected using the same scaling factor or the same correction coefficients without consideration of the disparities between the series to be corrected. Another weakness is that the statistical profile of the precipitation series is considered without accounting for the temporal profile. All these difficulties limit the correction of SPPs on the local scale.

Here, we propose to correct daily SPP estimates (TRMM-TMPA 3B42V7) using a novel two set approach. The first step defines hydroclimatic areas using a spatial classification that considers precipitation data with the same temporal distributions. The second step uses the Quantile Mapping bias correction method to correct the daily SPP data contained within each hydroclimatic area, by defining a calibration set and a validation set.

To identify the influence of the analysis scale on the statistical results, three simulations were conducted for the Guiana Shield using 93 daily rain gauges. The first simulation was performed for the entire study area, i.e., without any hydroclimatic division. The second simulation was undertaken with the study area divided into six hydroclimatic areas. The third simulation was performed with 23 hydroclimatic areas. For each simulation, we compared both the daily precipitation estimated from TRMM-TMPA 3B42V7 and the daily rain gauge measurements; and the precipitation corrected using the QM method and daily rain gauge measurements. We then validated the accuracy of the correction based on the RMSE and bias.

The remainder of this article is structured as follows: Section 2 describes the study area and presents the data used. Section 3 provides an overview of the methodology used to correct the biases. The results are presented and discussed in Section 4. Finally, the conclusions are drawn in Section 5.

## 2. Data

### 2.1. Study Area

**Figure 1:**
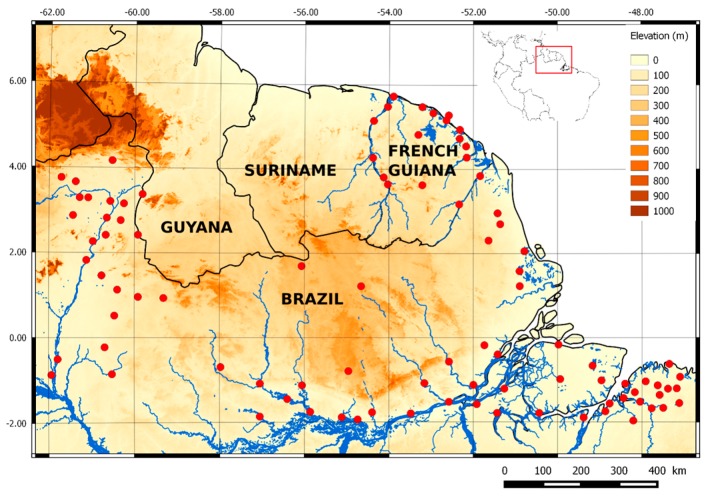
Elevation map of the Guiana Shield. The SRTM30 (Shuttle Radar Topography Mission) digital elevation model is available at http://www.diva-gis.org/gdata. Dots represent the daily rain gauges available in French Guiana and northern Brazil.

The Guiana Plateau, also called the Guiana Shield, is a region in South America located north of the Amazon River and east of the Orinoco River. This area is over 2 million km^2^. It spans six countries: Colombia, Venezuela, Guyana, Suriname, French Guiana, and northern Brazil (Amapá, Roraima, and Pará). The Guiana Shield has poor soil, an extensive river system, and dense primary rainforest [53]. This area accounts for 13% of the surface of the South American continent. In this study, the area considered lies within 2° S–6° N, 45°–62° W (Figure 1). The Guiana Shield is a region with high spatial variability in precipitation [11,54]. The annual average difference in precipitation between the littoral zone and inland areas can reach 2300 mm/year [11]. It is an area subject to intense and local convective precipitation. The network of precipitation measurements is very sparse and located primarily on the coast and along the rivers. Most of the present population lives on the coast or along the rivers and thus, is highly vulnerable to flooding.

### 2.2. Rain Gauges

The daily rain gauge data used in this study come from 93 daily rain gauges distributed between French Guiana and northern Brazil (Figure 1), including 18 in the Guyanese territory. North Brazilian data come from the Brazil National Water Agency (ANA). They are freely available online [55]. Rainfall data from French Guiana are provided by Météo France. To have the most complete time series and compare the SPP data, we use the period 2001–2012. The data are checked for quality and used in the following analysis.

### 2.3. Precipitation Product

A recent study by Ringard et al. [11] compared different SPPs in the Guiana Shield (TRMM-TMPA 3B42RT, TRMM-TMPA 3B42V7, PERSIANN, and CMORPH). The results obtained show that for areas with intense convective precipitation, TRMM-TMPA 3B42V7 performs better than the other products, especially in the estimation of extreme precipitation events. In regions along the Amazon, the use of PERSIANN is better. Finally, in the driest areas, TRMM-TMPA 3B42V7 and PERSIANN exhibit the same level of performance.

The daily SPPs used in this study come from the TRMM TMPA 3B42 algorithm, which was developed by NASA. A brief description of the TRMM-TMPA 3B42V7 product is given below. TRMM TMPA 3B42 is a rainfall estimation product from the TRMM mission that combines satellite and ground data [3,4,5,22,56,57]. The main data sources for TRMM-TMPA 3B42V7 are infrared GOES-W (Geostationary Operational Environmental Satellite-West), GOES-E (East), GMS (Geostationary Meteorological Satellite), Meteosat-5, Meteosat-7, and NOAA-12 geostationary satellites, as well as the passive microwave radiometers of the TMI/TRMM (TRMM Microwave Imager), SSMI/DMSP (Special Sensor Microwave Imager/Defense Meteorological Satellite Program), AMSU/NOAA (Advanced Microwave Sounding Unit/National Oceanic and Atmospheric Administration) and AMSR-E/Aqua (Advanced Microwave Scanning Radiometer-EOS) low orbit satellites.

During the last ten years, the TMPA algorithm has undergone three important updates to incorporate data from new sensors into the algorithm [22]. TRMM-TMPA 3B42V7, the research version, is available approximately two months after observation. The 3B42 algorithm runs in four stages [13]: (1) passive microwave precipitation estimates are calibrated and combined, (2) infrared precipitation estimates are generated using the calibrated data from the passive microwave sensors, (3) the infrared and passive microwave data are combined, and (4) the data are rescheduled on a monthly basis using the rain gauge data. Furthermore, TRMM-TMPA 3B42 uses precipitation estimates directly from the passive microwave data when available, but it inserts infrared data when the passive microwave data are unavailable [10]. TRMM-TMPA 3B42V7 algorithm output data have a 3 h time resolution with rainfall amounts expressed in mm/h. The geographical area covered extends from latitude 50° N–50° S for 3B42V7 with a 0.25° × 0.25° spatial grid resolution. The TRMM-TMPA 3B42V7 product data have been available since January 1, 1998 (through to the present day) [58].

## 3. Methods

We propose to correct daily TRMM-TMPA 3B42V7 estimates using a two set approach without taking into account the daily rain gauge data of the pixel to be corrected, but instead using the daily rain gauge data of the surrounding pixels. In this case, a spatial analysis must be involved. This novel set approach is divided into two steps: (1) several scales are defined through the definition of hydroclimatic zones; (2) bias correction using the Quantile Mapping approach is parameterized with the daily data contained in each hydroclimatic area, through the definition of a calibration set and a validation set.

### 3.1. Definition of Hydroclimatic Area

The aim is to identify the influence of the analysis scale on the efficiency of the Quantile Mapping correction method. For this purpose, we define hydroclimatic areas obtained from the rain gauge precipitation series based on long-term monthly means of the rain gauge data. The hydroclimatic areas are constructed using a hierarchical ascendant classification (HAC) [59,60]. The purpose of the classification approach is to obtain groups of rainfall time series with similar profiles. The creation of such groups makes it possible to distribute the precipitation series into different hydroclimatic areas.

At the initial stage of the method, each rain gauge forms a class, which makes 93 classes. The method proceeds by reducing the number of classes. At each step, two classes are grouped; thus, reducing the number of classes. The two classes chosen for grouping are those whose dissimilarity is the weakest; this dissimilarity value is called the aggregation index. Here, the aggregation index uses the centers of gravity of the classes, as in the Ward method [59].

**Figure 2:**
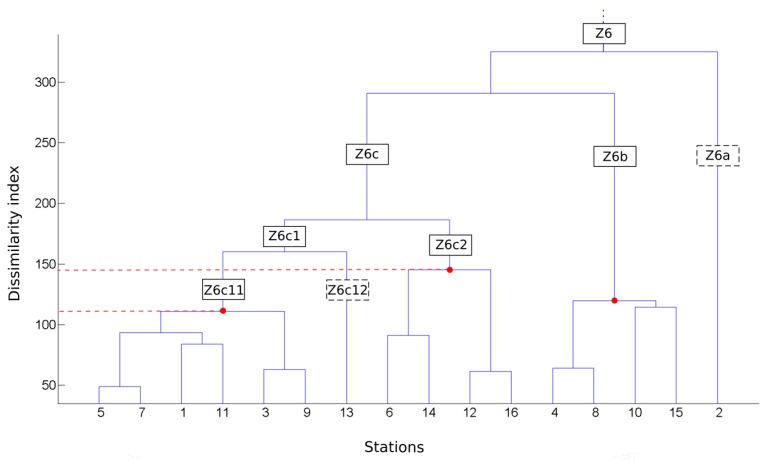
Diagram of the hierarchical ascendant classification for zone 6. Red dots indicate the dissimilarity values of the hydroclimatic groups conserved. Black squares in solid lines are the names of the areas at different hierarchical levels. Dotted black squares are classes with only one rain gauge, which are therefore unusable.

**Figure 3:**
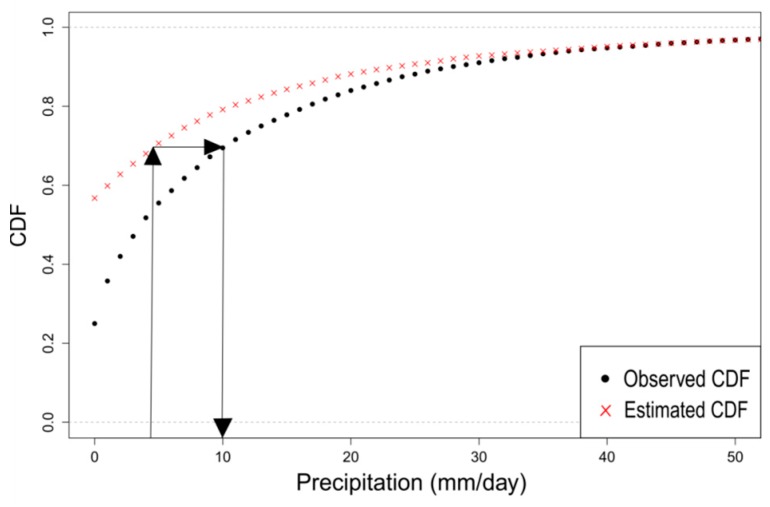
Schematic of the Quantile Mapping method. The distribution function of the SPP data is shifted to the distribution function of the rain gauge data.

Three simulations have been implemented to account for the influence of scaling analysis. The first simulation was performed on a single area, representing all the 93 rain gauges (i.e., the entire study area). The second simulation was performed on six classes representing six hydroclimatic areas. The third simulation was undertaken on 23 classes representing 23 hydroclimatic areas. The second simulation, carried out on six hydroclimatic areas, is the continuation of the work developed in Ringard et al. [11], who perform a regional analysis of SPPs in these six areas. Figure 2 shows an example of the classification performed in this study for zone 6 only. In the example shown in Figure 3, three areas are created from zone 6: Z6c11, Z6c2, and Z6b. The 23 hydroclimatic areas are obtained by following this same scheme.

### 3.2. Principles and Implementation of the Quantile Mapping (QM) Method

The second step uses the Quantile Mapping bias correction method to correct the daily SPP data contained within each hydroclimatic area, by defining a calibration set and a validation set. The QM method adjusts the distribution of daily satellite precipitation (*P_s_*) with the distribution of daily rain gauge precipitation (*P_o_*) using a transfer function (*h*). Figure 3 presents a schematic of the QM method. The transformation can be formulated as below [61,62]:

(1) $$P_{o}=h\left(P_{s}\right)$$

If the variable of interest has a known distribution, the transformation is defined as:

(2) $$P_{o}=F_{o}^{-1}\left(F_{s}\left(P_{s}\right)\right)$$

where *F_s_* is the Cumulative Distribution Function (CDF) of *P_s_* and $F_{o}^{-1}$ is the inverse CDF of *P_o_*.

There are several statistical transformations related to the QM method for modeling the quantile-quantile relationship [62]. The distribution-derived transformation uses a theoretical distribution to solve Equation (2). Parametric transformations are used directly to model the quantile-quantile relationship (Equation (1)). Finally, instead of assuming parametric distributions, nonparametric transformations use empirical CDFs to solve Equation (2) or nonparametric regressions such as cubic smoothing splines to solve Equation (1). Several approaches are possible; here, a smoothing spline is used to fit the quantile-quantile plot of daily observed and daily modeled time series. We relied on the work of Gudmundsson et al. [62] to implement this method.

#### Calibration of the QM Method and Correction of SPP Time Series

**Figure 4:**
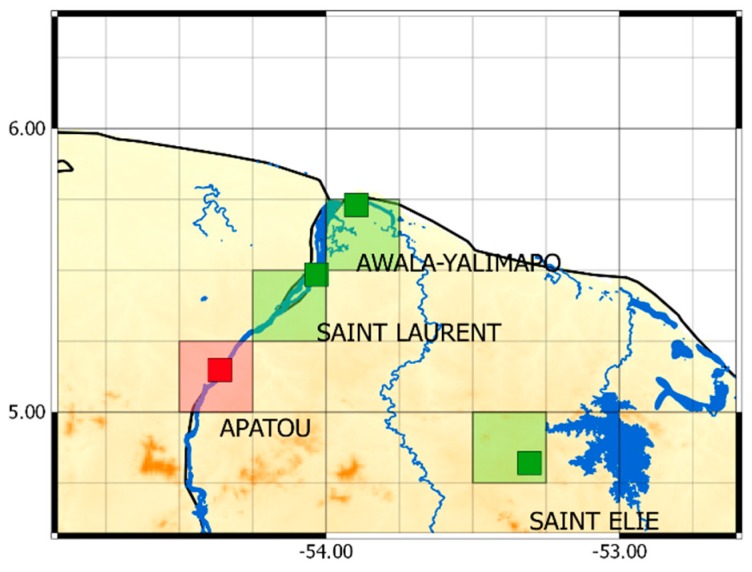
Diagram showing the calibration set (green) and validation set (red) for a hydroclimatic zone of four daily rain gauges with the TRMM-TMPA 3B42V7 grid.

To implement the QM correction method we divided each hydroclimatic area into two parts: calibration set and validation set. We take the example of a hydroclimatic area composed of four rain gauges represented by four pixels (Figure 4).

The calibration set is shown in green and the validation set in red. The calibration process uses the calibration set to adjust the distribution of the daily SPP data to match the distribution of the daily rain gauge data [62]. A function is set to adjust the precipitation data from a rainfall threshold set at 1 mm/d. The cut-off threshold is used to remove low precipitation values in the SPP data in order to equalize the frequency of wet days between the daily rain gauge and SPP precipitation data sets [63]. The result obtained corresponds to correction coefficients, calculated, for each percentile. The greater the number of quantile divisions used to represent the underlying frequency distributions, the better the correction [64]. The correction coefficients are calculated from the daily mean rain gauge series and the daily mean satellite series of the calibration set. In a second step, the correction coefficients obtained from the calibration set are applied to correct the SPP series of the validation set (red pixel in Figure 4). We implemented the same leave-one-out cross validation used in the paper of Kim et al. [63]: correction coefficients are calculated over 11 years and then applied to correct one year (the omitted year) of the SPP series of the validation set. This procedure is repeated for every year. The 12 years obtained by the validation process are combined into a single time series that forms the corrected SPP series.

In the example shown in Figure 4, the correction coefficients obtained from the calibration set (green pixels) are applied to the SPP series of the validation set (red pixel) to obtain the corrected SSP series. In each simulation on the 93 daily SPP series representing the available rain gauges, 70 daily SPP series are used as the calibration set, and 23 are selected as the validation set for the QM correction. The 23 daily SPP series of the validation set are chosen by selecting one rain gauge in each of the 23 hydroclimatic areas and the rest are used as calibration set. Comparison of the relative bias (rBIAS) and the relative RMSE (rRMSE) between the different simulations of the spatial clustering is performed on these same validation set.

## 4. Results

For each of the three simulations undertaken, we compared both the TRMM-TMPA 3B42V7 precipitation estimates against the daily rain gauge measurements, and the TRMM-TMPA 3B42V7 precipitation estimates corrected with the QM method against the daily rain gauge measurements.

The accuracy of the QM correction method in the various configurations presented above was evaluated via the calculation of the deviations: estimated – measured, and using statistical indices: rBIAS and rRMSE (Table 1).

### 4.1. Quality of Corrected TRMM-TMPA 3B42V7 Estimates for the Entire Study Area as a Calibration Set

In the first simulation, the parameterization of the QM correction method is performed for the entire zone using data from the 70 rain gauges in the calibration set.

#### 4.1.1. Global Assessment

**Figure 5:**
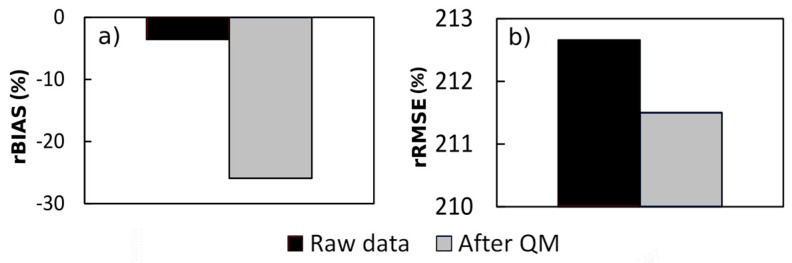
(**a**) Global relative bias (rBIAS; %) and (**b**) global relative RMSE (rRMSE; %) obtained by comparing the precipitation estimated from TRMM-TMPA 3B42V7 with daily rain gauge measurements (black), and the precipitation corrected with the QM method with daily rain gauge measurements (grey) over the entire study area.

Figure 5 shows the global rBIAS and global rRMSE for the 23 validation pixels considered as a whole. The biases and RMSEs are obtained after comparison of both the precipitation estimated from TRMM-TMPA 3B42V7 and daily rain gauge measurements (black), and the precipitation corrected with the QM method and daily rain gauge measurements (grey).

Figure 5a shows that the global rBIAS is worse after the QM correction. Without correction, the average of the rBIAS of the 23 daily SPP series is −3% against −25% after the QM correction. For the rRMSE, Figure 5b shows a very slight improvement of only 1% for the QM-corrected data. This result shows that for the Guiana Shield, the TRMM-TMPA 3B42V7 SPP provides a reasonably good estimation of precipitation on the global scale. The rBIAS degradation of the QM-corrected data indicates that this method is not applicable to large spatial scales.

#### 4.1.2. Local Assessment

**Figure 6:**
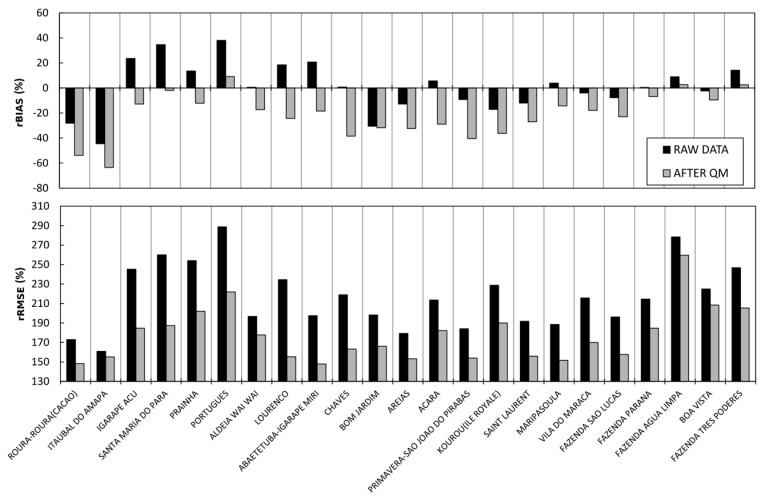
Relative bias (rBIAS; %) and relative RMSE (rRMSE; %) obtained by comparison of both the precipitation estimated from TRMM-TMPA 3B42V7 and daily rain gauge measurements (black), and the precipitation corrected with the QM method and daily rain gauge measurements (grey) for the validation pixel in each of the 23 hydroclimatic areas.

Figure 6 shows the rBIASs and rRMSEs for the 23 validation daily SPP series considered individually. The rBIASs and rRMSEs are obtained after comparing the precipitation estimated from TRMM-TMPA 3B42V7 and daily rain gauge measurements (black), and the precipitation corrected with the QM method and daily rain gauge measurements (grey).

Figure 6 shows that only four daily SPP series have a low bias without applying the QM correction. When the QM correction is applied throughout the entire study area, seven of the 23 validation daily SPP series show a correction in rBIAS and 16 daily SPP series show a deterioration in rBIAS in comparison with the raw data. For the seven daily SPP series corrected, the improvement of rBIAS is on average 13.4%. Conversely, for the 16 uncorrected daily SPP series, the deterioration of rBIAS is on average 16.7%. Regarding the rRMSE, after the QM correction, the 23 daily SPP series show lower rRMSEs with an average improvement of 40%. These results show that for the Guiana Shield, the QM correction increases the bias for a large number of daily SPP series and thus, it is not a method that is applicable to large spatial scales.

### 4.2. Quality of Corrected TRMM-TMPA 3B42V7 Estimates for 6 Hydroclimatic Areas as Calibration Sets

In the second simulation, we apply the HAC method to all the rain gauge data to obtain 6 classes of hydroclimatic regime. The QM correction method is then parameterized with data contained in each hydroclimatic regime. The parameterization of the QM correction method is done for each of the 6 hydroclimatic zones, and these parameters are applied to the validation pixels located in each of the hydroclimatic zones.

**Figure 7:**
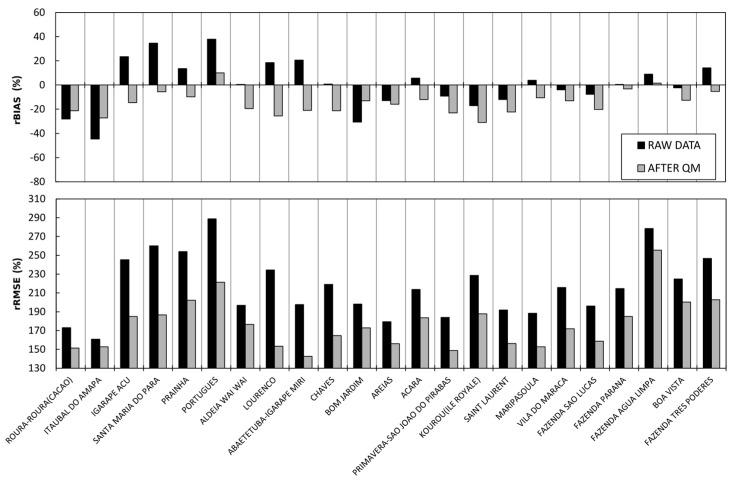
(**a**) Relative bias (rBIAS; %) and (**b**) relative RMSE (rRMSE; %) obtained by comparison of both the precipitation estimated from TRMM-TMPA 3B42V7 and daily rain gauge measurements (black), and the precipitation corrected with the QM method and daily rain gauge measurements (grey) for 23 pixels in 6 hydroclimatic areas.

Figure 7 shows the rBIASs and rRMSEs for the validation daily SPP series. The rBIASs and rRMSEs are obtained by comparing both the TRMM-TMPA 3B42V7 precipitation estimates and daily rain gauge measurements, and the TRMM-TMPA 3B42V7 precipitation estimates corrected with the QM method and daily rain gauge measurements.

In comparison with the raw data, nine of the 23 validation daily SPP series show a correction in rBIAS and 14 daily SPP series show a deterioration in rBIAS. The improvement of rBIAS for the nine validation daily SPP series is on average 14.2%. The deterioration of rBIAS for the other 14 validation daily SPP series is on average 9.7%. Compared with the first simulation, it is evident that only two more daily SPP series show an improvement after QM. The improvement of the nine validation daily SPP series is slightly better than in the previous simulation. However, the degradation of the 14 daily SPP series after the QM correction is much less significant when taking into account the six hydroclimatic zones rather than considering the entire region as a single hydroclimatic area.

Regarding the rRMSEs, after QM correction, 23 daily SPP series show lower rRMSEs with an average improvement of 40%. The improvement is of the same order of magnitude as the first simulation. These results show that for the Guiana Shield, the correction by QM shows a bias degradation for a large number of daily SPP series and that it is therefore not applicable to large spatial scales.

These results show that dividing the study area into six different hydroclimatic areas and correcting the bias using the parameterized QM correction method with the data contained in each of the hydroclimatic zones makes it possible to improve the rBIAS of the TRMM-TMPA 3B42V7 SPP slightly. However, more than half the daily SPP series remain uncorrected. On the regional scale, the TRMM-TMPA 3B42V7 SPP shows greater difficulty in correctly estimating precipitation. Although the QM correction makes it possible to correct some regions, this correction method still has difficulties in improving the bias in certain regions.

### 4.3. Quality of corrected TRMM-TMPA 3B42V7 Estimates for 23 Hydroclimatic Areas as Calibration Sets

**Figure 8:**
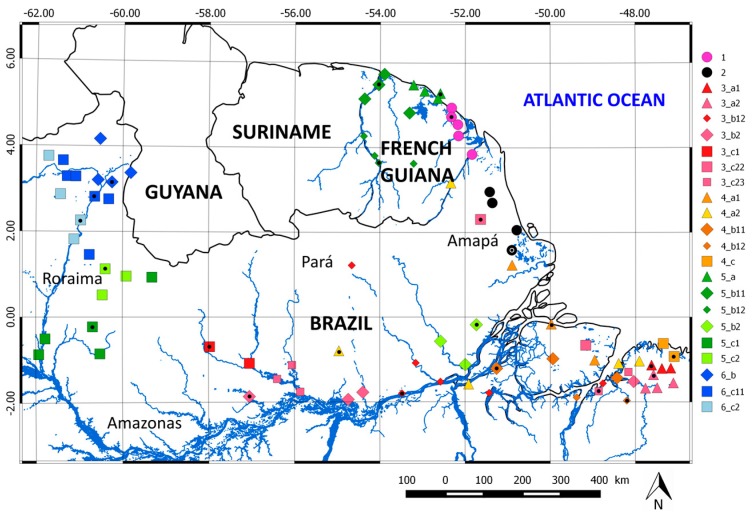
Spatial map of the 23 hydroclimatic areas obtained by the hierarchical ascendant classification method. Black dots represent the validation pixels.

In this third simulation, the QM correction method is applied to the 23 hydroclimatic areas obtained by the HAC method and represented in Figure 8. For each of the 23 hydroclimatic areas, one pixels is used as the validation daily SPP series. The remaining pixels in each area are used as calibration daily SPP series for the QM correction method.

**Figure 9:**
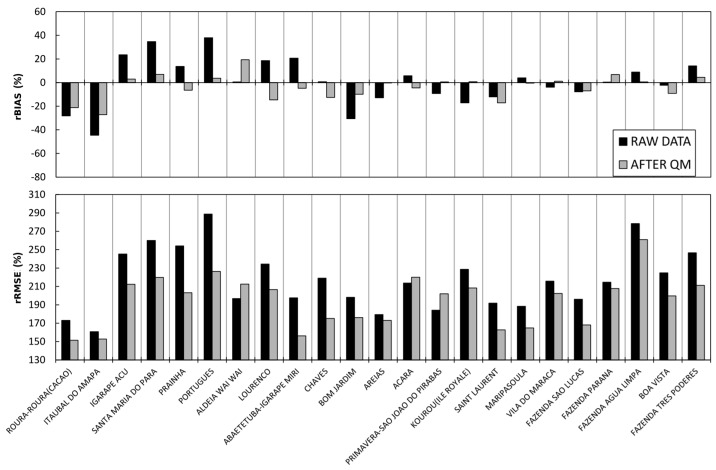
(**a**) Relative bias (rBIAS; %) and (**b**) relative RMSE (rRMSE; %) obtained by comparison of both the precipitation estimated from TRMM-TMPA 3B42V7 and daily rain gauge measurements (black), and the precipitation corrected with the QM method and daily rain gauge measurements (grey) for 23 pixels using 23 hydroclimatic areas.

The parameterization of the QM correction method is done for each of the 23 hydroclimatic areas, and these parameters are applied to the validation daily SPP series located in each hydroclimatic area. The rBIASs and rRMSEs are calculated by comparing the satellite data with the QM corrected data (Figure 9). Figure 9 shows the results of the rBIASs and rRMSEs for each of the 23 validation daily SPP series. Compared with the raw satellite data, 18 of the 23 validation daily SPP series show a correction of rBIAS and only five daily SPP series show a worsening of rBIAS. The improvement of rBIAS for the corrected daily SPP series is on average 12.1%. The deterioration of rBIAS for the degraded daily SPP series is on average 9.8%. Compared with the first two simulations, the improvement in rBIAS is much better, with 78% of validation daily SPP series showing a correction of rBIAS after QM correction.

Regarding the rRMSEs, after QM correction, 20 daily SPP series show lower rRMSEs with an average improvement of 28%. Only three daily SPP series show larger rRMSEs after QM correction.

### 4.4. Performance

**Figure 10:**
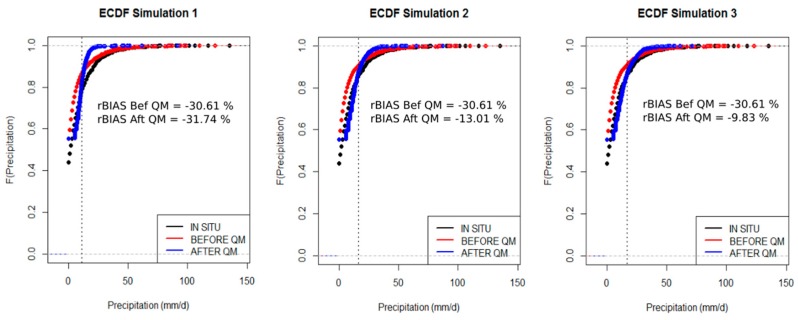
Empirical Cumulative Distribution Function for a rain gauge and its associated pixel under the three simulations. Each simulation shows the ECDF of the daily rain gauge (black), the ECDF of SPP before QM (red) and the ECDF of SPP after QM (blue).

Figure 10 shows the Empirical Cumulative Distribution Function (ECDF) of daily rain gauge data, SPP data and of corrected SPP data for the three simulations under consideration. In the first simulation the parameterization of the QM correction method is done for the entire zone with the data from the 70 rain gauges in the calibration set. The ECDF of the corrected SPP shows no improvement and seems to be moving away from the ECDF of the daily rain gauge data, in particular for intensities greater than 15 mm/d. The relative bias of the corrected SPP (−31.74%) is about 1% greater than the relative bias before QM correction (−30.61%). For intensities less than 15 mm/d, i.e., 80% of the recorded precipitation, the correction is remarkably effective. However, for intensities between 15 and 40 mm/d, the ECDF of the corrected SPP data is largely greater than the gauge’s ECDF. High intensities are not corrected.

In the second simulation, the QM correction method is parameterized with data from each of the six hydroclimatic regimes. The relative bias of the SPP data improved markedly from −30.61% before QM correction to −13.01% after QM correction. For intensities less than 20 mm/d, i.e., about 85% of precipitation, the QM correction is effective. For intensities between 20 and 50 mm/d, the ECDF of the SPP after correction is still a little removed from the ECDF of the daily rain gauge measurement.

In the third simulation, the QM correction method is parameterized with data from each of the 23 hydroclimatic regimes. The ECDF of the corrected SPP data shows better agreement with the daily rain gauge compared to the ECDF of SPP data before QM correction. Indeed, the relative bias is improved from −30.61% to −9.83%. For intensities less than 25 mm/d, i.e., about 90% of precipitation, the QM correction shows very good results. However, the tendency to correct the intensity between 25 and 50 mm/d is more difficult.

In summary, the QM method corrects the weak and high intensities rather than the medium intensities. The results show this novel two set approach reduces bias and RMSE significantly.

## 5. Discussion and Conclusions

The objective was to demonstrate the importance of considering hydroclimatic regimes in the correction of daily satellite precipitation data. We used a spatial classification approach and exploited the QM method to correct daily TRMM-TMPA 3B42V7 SPP estimates. The rain gauge measurement pixels were grouped spatially into different hydroclimatic zones according to their temporal profiles based on long-term monthly means of precipitation data using the HAC method. Then, parameterization of the QM method was performed for each of the hydroclimatic areas, and these parameters were used to correct the daily TRMM-TMPA 3B42V7 SPP estimates. In order to show how variation in the scale of analysis affected the rBIAS and rRMSE, three simulations with different spatial divisions were conducted, and the effects of the QM method on the accuracy of the corrected SPP estimates were observed.

For the global scale (simulation 1), applying the QM correction TRMM-TMPA 3B42V7 has a poor effect: only seven out of 23 daily SPP series are corrected. The approach used in this paper, which applies the correction to the satellite series from the calibration daily SPP series throughout the zone, does not make it possible to correct the bias present in the SPP. The QM correction method, on a global scale, is limited in its ability to correct atmospheric phenomena that occur at finer scale. Even after reducing the spatial scale to 6 hydroclimatic groups (simulation 2), the QM correction applied within each hydroclimatic area still shows significant relative biases. Only nine out of 23 daily SPP series have an improved relative bias after the QM correction. However, the daily SPP serie that are not corrected have smaller errors than in simulation 1. In order to improve the relative bias, we once again reduced the spatial scale in order to correct daily SPP series within a smaller area that is characteristic of a more precise climate regime. Simulation 3 divides the area into 23 hydroclimatic groups. At this finer scale, bias results are significantly improved. Of the 23 daily SPP series, which correspond to the 23 areas, 18 show an improvement in rBIAS, and 20 show an improvement in rRMSE. Applying the QM correction method to the data contained in each hydroclimatic area makes it possible to improve the accuracy of the TRMM-TMPA 3B42V7 SPP at the finer spatial scale.

The principal finding was that the QM bias correction performed worst at the coarsest scale, whereas additional benefits were produced at a finer scale. Classifying the rain gauge data by hydroclimatic area clearly improved the rBIASs and rRMSEs, reducing errors in the precipitation estimates. The temporal characteristics of the sampling units, such as amplitude or similarity of the time series, are important scale concepts. The analysis of the ECDF shows that the QM method corrects the weak intensities rather than the medium intensities.

It is important to note a deterioration of rBIAS after QM correction for certain SPP series. The daily SPP series with a degradation of rBIAS can be classified according to two cases. The first case are SPP series that considerably overestimate or underestimate precipitation before QM correction, which is amplified after QM correction. The second case is SPP series that have low rBIAS before QM correction, which see their rBIAS degraded after QM correction. This category of SPP series does not require QM correction because the satellite series is very close to the gauge series. For example, within the 23 hydroclimatic areas, only five daily SPP series showed degradation. Of these five, three showed rBIASs before correction of <1%. Therefore, only two daily SPP series showed a large degradation of rBIAS.

A multitude of studies show that the performance of SPPs is a function of precipitation intensities, climate and seasons. Simulation 1 calibrates the correction method from 70 rain gauges available throughout the region. Calibration is therefore carried out on pixels which may have a precipitation difference of more than 2000 mm/year. Even after subdividing the area into 6 regions, (Simulation 2), the correction’s effectiveness is evidently still limited. The more we decompose into hydroclimatic zones, the more we group together, within a zone, rain gauges that are very close climatically. This grouping improves the efficiency of the QM correction to reduce the bias. Consequently, if the areas that are used to define the correction parameters contain more homogeneous time data, the more likely it is that the quality of the correction is high. This is because the correction will apply to a SPP series with a time profile “similar” to those used for the calibration of the correction parameters. Conversely, if the correction parameters are established from areas containing more heterogeneous data, it is more likely that the correction parameters will be poorly adapted to the SPP series to be corrected, and hence the quality of the correction will be of low quality. This shows the importance of taking into account spatial scale in the QM correction method.

Several hypotheses may be invoked to explain this degradation. A first hypothesis is that the method compares and corrects a satellite pixel covering 625 km^2^ with a rain gauge point. There is therefore an incompressible bias between the amount of precipitation at the rain gauge point and the amount of precipitation in the satellite pixel. This is linked to localized phenomena such as convective rain, which can be very intense and spatially restricted. A second hypothesis is related to the criterion used to define the hydroclimatic zones, i.e., the 12 monthly averages of precipitation data. This criterion can lead to bias degradation linked to different temporal phenomena existing during the 12 years of the time series, but it can also lead to identical hydroclimatic regimes when monthly averages are used.

From a spatial perspective, studies have been carried out on the capacity of SPP to estimate precipitation in mountainous areas. Zambrano-Bigiarini et al. [65] compare 14 regions across Chile and show that SPPs perform better in low and mid-altitude regions. In our study, the altitude of the 23 daily rain gauges associated to the 23 validation daily SPP series varies from 1 m (Chaves and Kourou) to 305 m (Lourenco). We observe, with the third simulation, that among the five lowest daily rain gauges and associated SPP series (>10 m), only one sees its bias increase after QM correction. Conversely, the five highest daily rain gauges and associated SPP series (>100 m), all show a decrease in bias after QM correction in the associated daily SPP series. The five daily SPP series that see their bias deteriorate after QM correction, in simulation 3, are at low and mid-altitudes of 1, 12, 16, 71 and 76 m. These results show that the topography of our region does not explain the worsening of the bias. The second possible explanation of the bias differences after correction is distance to the coast. Among the five daily SPP series that show a deterioration of the bias after correction in the third simulation, the distances to the coast are very variable. The validation daily rain gauge in the area 5c2 (Fazenda Parana) is 640 km from the coast, while the validation daily rain gauge in the areas 5b11 (Saint Laurent) and 4a1 (Chaves) are located at a distance of just 20 km and 2 km, respectively. The distance to the coast cannot therefore be used as a criterion to justify the degradation at these daily SPP series. In terms of land cover, of our 23 validation daily rain gauge associated to daily SPP series, 7 are at a distance of less than 1 km from the lake or river, of which four show a deterioration of the bias after correction, i.e., more than half. In the microwave domain, the radiometer receives the signal of the diffusion and absorption of hydrometeors but also of the terrestrial surfaces, thus measuring the moisture content of these surfaces. Our results may therefore show that the proximity of water has an influence on the difficulty of the SPP in the estimation of the precipitations and thus the difficulty of correctly correcting certain daily SPP series. The radiometer measures moisture content above water bodies and thus overestimates precipitation. These results are confirmed by Delahaye [8] which observes errors of estimation over dense forests, due to the high moisture content captured by the radiometer.

In addition to a spatial approach, a temporal approach could be considered. In other words, the satellite data could be corrected according to season or according to other criteria such as the temporal characteristics of rainfall [63]. This would make it possible to divide the time series into several time sequences, and to correct each of the sequences according to different correction coefficients.

Our study has indicated that hydroclimatic classification is relevant for establishing a bias correction method. We conclude that this novel two set approach to correct for TRMM-TMPA 3B42V7 SPP estimates provides acceptable results for the Guiana Shield. The perspective ultimately aims to use these results to design a methodology for correcting SSP pixels on a wider scale with a limited amount of gauge data. Future work will investigate the possibility of correcting TRMM-TMPA 3B42V7 SPPs based on predefined hydroclimatic areas. Thus, each satellite pixel will be associated with a hydroclimatic regime and will be corrected based on the rain gauge data present within the same hydroclimatic regime. This work will make it possible to improve the correction of TRMM-TMPA 3B42V7 SPPs at the large scale for areas with a sparse rain gauge measurement network.

## Figures and Tables

**Table 1:** Quantitative statistical criteria. For a given rain gauge and its associated pixel, O_i_ is the rain gauge value and E_i_ the satellite value. N is the total number of days in the time series.

Statistical Criteria	Formula
BIAS	$\frac{1}{N}\;\sum_{i=1}^{N}(E_{i}-O_{i})$
rBIAS	$\frac{Biais}{\bar{O}}$
RMSE	$\sqrt{\frac{1}{N}\;\sum_{i=1}^{N}{\left(E_{i}-O_{i}\right)}^{2}}$
rRMSE	$\frac{RMSE}{\bar{O}}$
